# jdispatcher-viewers: interactive visualizations of sequence similarity search results and domain predictions

**DOI:** 10.1093/bioadv/vbaf122

**Published:** 2025-05-23

**Authors:** Fábio Madeira, Joonheung Lee, Nandana Madhusoodanan, Alberto Eusebi, Ania Niewielska, Sarah Butcher

**Affiliations:** European Molecular Biology Laboratory, European Bioinformatics Institute (EMBL-EBI), Wellcome Trust Genome Campus, Hinxton, Cambridge CB10 1SD, United Kingdom; European Molecular Biology Laboratory, European Bioinformatics Institute (EMBL-EBI), Wellcome Trust Genome Campus, Hinxton, Cambridge CB10 1SD, United Kingdom; European Molecular Biology Laboratory, European Bioinformatics Institute (EMBL-EBI), Wellcome Trust Genome Campus, Hinxton, Cambridge CB10 1SD, United Kingdom; European Molecular Biology Laboratory, European Bioinformatics Institute (EMBL-EBI), Wellcome Trust Genome Campus, Hinxton, Cambridge CB10 1SD, United Kingdom; European Molecular Biology Laboratory, European Bioinformatics Institute (EMBL-EBI), Wellcome Trust Genome Campus, Hinxton, Cambridge CB10 1SD, United Kingdom; European Molecular Biology Laboratory, European Bioinformatics Institute (EMBL-EBI), Wellcome Trust Genome Campus, Hinxton, Cambridge CB10 1SD, United Kingdom

## Abstract

**Motivation:**

Biological visualization is an important technique for researchers to make sense of complex biological data. Functional prediction and the discovery of novel proteins remain central objectives in biology, as they provide insights into molecular mechanisms with significant applications in health and disease. Visualizing sequence similarity search results and domain predictions is essential for exploring protein function, identifying conserved elements, and drawing meaningful connections between sequences, ultimately accelerating discovery.

**Results:**

The new website for the EMBL-EBI Job Dispatcher bioinformatics tools framework, was released in 2023. Along with improvements and new features, the website has since integrated interactive visualizations designed to aid researchers further and enrich the user experience. Here, we describe jdispatcher-viewers, a library for the interactive visualization of sequence similarity search results from BLAST and FASTA, and interactive visualizations of domain predictions and annotations provided by InterPro.

**Availability and implementation:**

The jdispatcher-viewers library and documentation which includes a demo webpage are available from https://github.com/ebi-jdispatcher/jdispatcher-viewers. Interactive visualizations provided among the result pages of sequence similarity search tools in Job Dispatcher have been implemented using jdispatcher-viewers, and are available at https://www.ebi.ac.uk/jdispatcher/sss. The library is distributed under the Apache 2.0 license.

## 1 Introduction

Biological visualization is an important technique that helps researchers make sense of complex biological data (Gehlenborg *et al.* 2010). Visualization methods are continuously evolving and accompanying advances in the life sciences. From foundational works such as the early depictions of evolutionary trees (Gregory 2008) and diagrams of cellular pathways (Schreiber *et al.* 2022), to contemporary cartoon representations of 3D protein structures (Sehnal *et al.* 2021), there is currently a large variety of biological visualizations representing different data types and visualization needs. These are crucial not only for researchers as part of their toolbox but also for education and communication purposes, as seen in school textbooks and any recent biology journal. Technological advances have expanded visualization beyond paper, allowing for display across devices from smartphones to virtual reality.

The success of the web and the development of browsers with remarkable graphic capabilities has allowed the development of modern visualizations which are increasingly interactive (Hildebrand *et al.* 2019), allowing users to filter data, highlight and expand on areas of interest, and manipulate various visual elements. The web is therefore the prime platform for providing and disseminating data-rich biological visualizations. Its broad reach makes complex scientific data widely accessible and useful to researchers, educators, and the public. The web ecosystem provides standards, such as SVG, HTML5 canvas, and WebGL, that support the creation and rendering of high-quality, responsive visualizations. These standards ensure that complex visualizations can be displayed in real-time, consistently across browsers and devices, providing a reliable and smooth user experience.

The functional characterization of unknown proteins remains central in biology, as it provides insights into molecular mechanisms with significant applications in health and disease (Jaroszewski *et al.* 2009, Wood *et al.* 2019, Rodríguez del Río *et al.* 2024). Common functional prediction visualizations are domain structure diagrams, where each domain is displayed on a linear sequence track often represented as colour-coded bars. The position and length of each domain are meant to represent where the domain is found in the sequence space. Another useful visualization is the graphical overview of sequence similarity search (SSS) tool results. Following the same approach, sequence hits are displayed as coloured bars, in relation to the query sequence, highlighting conserved regions and the alignment confidence. Hits are usually ranked and colour-coded based on *E*-value or other scores such as sequence similarity. The visualization of SSS results and domain predictions are therefore a useful tool for exploring protein function, identifying conserved elements, and drawing meaningful connections between sequences, ultimately accelerating discovery.

The EMBL-EBI Job Dispatcher tools framework (Madeira *et al.* 2024) enables the scientific community to perform a diverse range of sequence analyses using widely used bioinformatics applications. The new Job Dispatcher website was released in late 2023, and among several new features and improvements, it provides user-friendly visualizations such as interactive multiple sequence alignments and phylogenetic trees, powered by Nightingale (Salazar *et al.* 2023) and phylotree.js (Shank *et al.* 2018), respectively. New interactive visualizations were developed for SSS results, particularly interactive graphical representations of sequence similarity search (SSS) tool outputs ‘Visual Output’ and ‘Functional Predictions’ provided by InterPro (Blum *et al.* 2021). These are custom-made and are available from the jdispatcher-viewers library, described in this paper.

## 2 Methods

jdispatcher-viewers is available as an ECMAScript (ES) Module and is written in TypeScript (https://www.typescriptlang.org/). Two main custom visualizations are currently provided: SSS Visual Output and SSS Functional Predictions. These are also provided as standalone HTML Web Components or can be included as plain JavaScript, embedded in the script tag elements of HTML pages. The library can be built as a Node v18.20 (https://nodejs.org/) command-line interface (CLI) application, useful for generating static images in SVG and PNG formats. Interactive visualizations are powered by Fabric.js v5.4.0 (https://fabricjs.com/). Fabric.js is a library built on top of the Canvas API, which simplifies working with HTML5 canvas. Fabric.js provides a rich set of tools for drawing shapes and text elements, exposed by an object-oriented API. jdispatcher-viewers is open source, and it is distributed under the Apache 2.0 license. The library is hosted on GitHub (https://github.com/ebi-jdispatcher/jdispatcher-viewers) and we welcome contributions from the community. Documentation is available from https://ebi-jdispatcher.github.io/jdispatcher-viewers/. The package is also published in npm (https://www.npmjs.com/package/@ebi-jdispatcher/jdispatcher-viewers), making it easy to integrate with JavaScript/TypeScript projects.

## 3 Results

**Figure 1.**
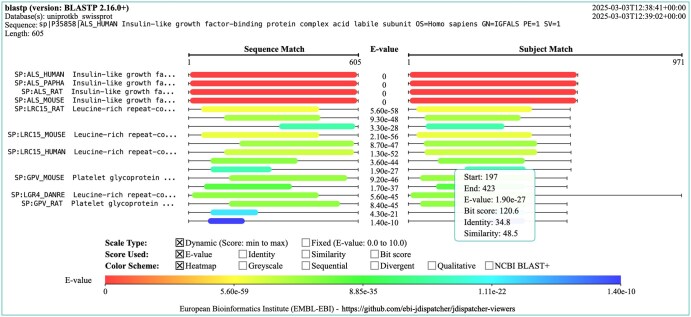
Example visual output visualization generated for the result of an NCBI BLAST+ blastp sequence search. The visualization header provides the tool and version, the database selected, the query sequence used, and the sequence length. The top right also shows the execution’s start and end timestamp. The central area of the visualization shows the query and subject alignment view, with the sequence range of the hit and subject alignments displayed as coloured bars. The left panel shows the sequence IDs of the hits displayed. The scale, score and colour applied can be selected by clicking on the checkboxes displayed above the scale bar. Hovering and clicking on the bars reveals a tooltip with information about the match.

**Figure 2.**
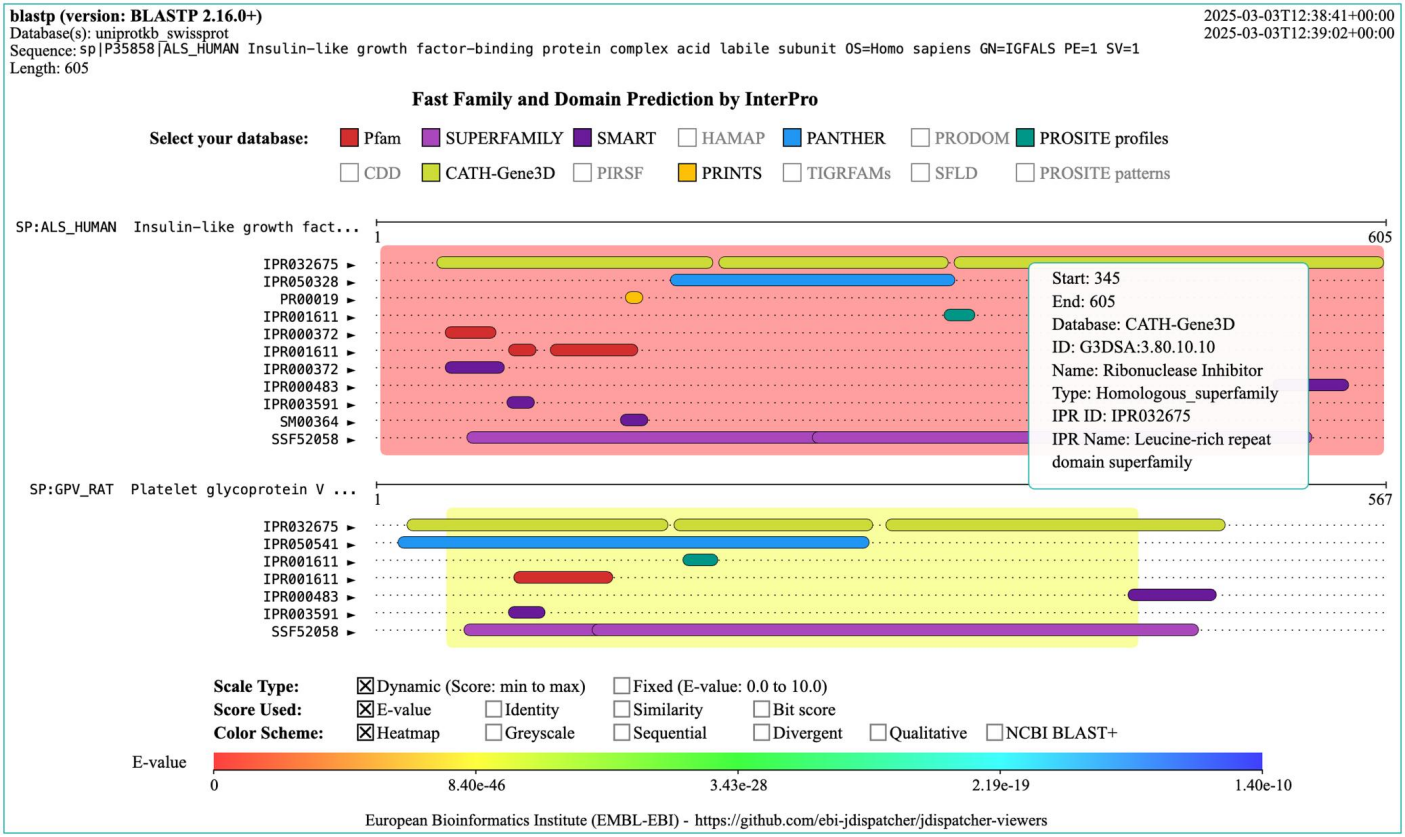
Example functional predictions visualization generated for the result of an NCBI BLAST+ blastp sequence search. Domain annotations and predictions are obtained from InterPro. The visualization header provides the tool and version, the database selected, the query sequence used, and the sequence length. The top right also shows the execution’s start and end timestamp. The central area of the visualization shows the subject alignment view, with the sequence range of the subject alignments displayed as coloured backgrounds. Domain bars are overlaid on top and are coloured based on the annotation source. These can be displayed or hidden on mouse clicking in the ‘Select your database’ panel entries. Some of the annotation resources are disabled if there is no annotation for that source in the results of the sequence search. The left panel shows the sequence IDs of the hits displayed. The scale, score and background colour applied can be selected by clicking on the checkboxes displayed above the scale bar. Hovering and clicking on the domain reveals a tooltip with information about the domain, its ID, name, resource name, etc.

The jdispatcher-viewers library provides two main visualizations for the results of SSS tools. These are provided by default in the result pages of Job Dispatcher. The two visualizations are (i) a graphical representation of the SSS tool results, referred to as the Visual Output visualization and; (ii) domain prediction diagram, referred to as the Functional Predictions visualization. See Figs 1 and 2, e.g. of these visualizations, respectively.

### 3.1 Visual output

In a typical SSS tool output such as those from NCBI BLAST+ (Camacho *et al.* 2009) or FASTA (Pearson and Lipman 1988), the alignment between the query and subject sequences, usually referred to as hit sequences, is typically shown as high-scoring segment pairs (HSPs), which represent regions of significant similarity. The graphical representation enables rapid identification of the highest-quality matches, assessing hit alignment coverage, and determining areas of local alignment. These visualizations reveal conserved regions across homologous sequences. The conserved segments often indicate functional or structural importance, such as active sites in enzymes or binding sites in receptors, providing insights into possible biological functions.

The Visual Output visualization is interactive (see Fig. 1). All sequence identifiers are clickable, as they provide links to the corresponding resource [e.g. UniProt (The UniProt Consortium 2023) and PDBe (Armstrong *et al.* 2020)]. Bars can display additional information on mouse hovering and clicking, particularly the sequence start and end ranges, as well as several scores, such as *E*-value and bit scores. The scale type can be toggled between ‘dynamic’, where the start-to-end range is based on the sample of scores found in the data; and ‘fixed’, where the start-to-end range is fixed and depends on the selected score (e.g. min.: 0 and max: 100, if the sequence identity is selected). The score being displayed can also be toggled between *E*-value, bit score, sequence identity and sequence similarity. Finally, the colouring scheme can also be toggled, the default being heatmapOther schemes are available including a sequential greyscale (grey hue), a sequential blue scale (blue hue), a divergent (red to green), a qualitative (contrasting colours), and lastly a qualitative colouring based on NCBI BLAST+ bit score ranges.

This visualization can be found in the ‘Visual Output’ tab on the Job Dispatcher SSS result pages. A static image can also be downloaded in PNG format from the same view.

### 3.2 Functional predictions

The Functional Predictions visualization combines the Visual Output visualization with domain diagrams (see Fig. 2). For each hit sequence match, domain predictions by InterPro (Blum *et al.* 2021) are provided. The scale, scoring and colouring scheme are selectable and are applied as described for the Visual Output visualization. The main difference here is that the hit bar is displayed in the background and domain annotation bars are overlaid on top. All domain predictions from various sources [e.g. Pfam (El-Gebali *et al.* 2019), SUPERFAMILY (Pandurangan *et al.* 2019), etc.] are displayed by default but these can be toggled between hiding and display states. This visualization can be found in the ‘Functional Predictions’ tab on the Job Dispatcher SSS result pages. A static image can be downloaded in PNG format.

### 3.3 Reusable components

The jdispatcher-viewers library exposes several constructs, including types, interfaces, classes and functions, alongside utilities that together with Fabric.js can be used to compose new custom visualizations:

- Custom types and interfaces—these are useful to define common visual elements such as rectangles, lines, text, and to define other objects such as render and coordinate options and default values. These are meant to work directly with Fabric.js data types and objects.
- Data models—these are useful to work with SSS JSON results (see https://github.com/ebi-jdispatcher/sss_json_schema), from NCBI BLAST+ and FASTA. These also define models for working with InterPro domain prediction annotations.
- Colour schemes and colouring functions—these are useful for applying a variety of colour schemes and creating colour gradients.
- Drawing and other utilities—these are useful as they enable building custom visualizations that follow the look and feel of the two main visualizations currently available. It also includes utilities to fetch results from Job Dispatcher and InterPro APIs, as well as, to draw several visual elements such as sequence tracks, domains, scale bars, etc.

Some of the current visualization defaults are configurable. The canvas width and height, e.g. can be edited, as can the font, font size, width of labels and contents, etc. The default colouring styles can also be configured. Lastly, the number of hits and HSPs to be displayed can be modified from the defaults which are 100 (10 HSPs) and 30, for Visual Output and Functional Predictions, respectively.

## 4 Discussion

The jdispatcher-viewers library provides two main visualizations that are integrated into the results pages of the Job Dispatcher sequence search tools. A few alternative software and web application solutions have been developed over the last years but generally do not provide similar visualization experiences. If we disregard, for comparison purposes, commercial, closed-source alternatives, and those that are no longer available to use, then only a handful of libraries can be compared. BlasterJS (Blanco-Míguez *et al.* 2018) works by parsing the NCBI BLAST+ tool output. It provides only two colouring schemes and a selection of either *E*-value or bit score. BlasterJS was developed as a BioJS library (Corpas *et al.* 2014) but offers limited customization and its source code has not been updated since 2019, making it not an option for the development of new visualizations. Nightingale (Salazar *et al.* 2023) is a library of reusable data visualization web components for protein feature visualization. It is still under active development and follows web standards but does not provide an out-of-the-box component for visualizing sequence search results. With some effort, one could theoretically compose similar-looking visualizations with its existing components. The ‘track’ and ‘interpro-track’ components could be used to display search hits as annotated segments on a query sequence, with colours indicating scores. This would require building a custom data model mapping and some other customizations. The NCBI BLAST+ (Camacho *et al.* 2009) ‘Graphic Summary’ visualization is not an alternative as the source code for the web interface’s graphical features is not part of the open BLAST+ release. It also lacks interactivity as no customizable viewing options or colouring schemes are provided. While some visualizations for NCBI BLAST+ results exist, we note the Functional Predictions visualization described here, is to our knowledge, unique, in the way it overlays domain predictions and annotations on top of the sequence search output. This enriches the interpretation of the sequence alignment matches in the context of known and predicted domains and their arrangement in the sequence space. One main limitation is the fact that jdispatcher-viewers rely on Job Dispatcher SSS JSON result files. These are used to populate the data model required by jdispatcher-viewers to display the various data elements. We aim to update the library so that it can load the sequence search tool outputs directly, which would improve the utility of the library and reach a broader group of users. We would like to further improve the abstraction and modularization of the library to increase its scope, particularly for developers aiming at developing new visualizations reusing the building blocks that the library implements. Notably, user and developer documentation is available, but we aim to expand the examples on how to build new custom visualizations with Fabric.js and jdispatcher-viewers.
